# Associations between the household environment and stunted child growth in rural India: a cross-sectional analysis

**DOI:** 10.14324/111.444/ucloe.000014

**Published:** 2021-02-22

**Authors:** Charlotte Lee, Monica Lakhanpaul, Bernardo Maza Stern, Kaushik Sarkar, Priti Parikh

**Affiliations:** 1 Whittington Health NHS Trust, Magdala Avenue, London N19 5NF, UK; 2 UCL Great Ormond Street Institute of Child Health, 30 Guildford Street, London WC1N 1EH, UK; 3 Engineering for International Development Centre, University College London, Bartlett School of Construction and Project Management, 1–19 Torrington Place, London WC1E 7HB, UK; 4 Aceso Global Health Consultants Limited, B-78-A FF Front Side, Chanakya Place–1, Uttam Nagar, New Delhi 110059, India

**Keywords:** interdisciplinary, environment, water, sanitation, agriculture, cooking fuel, malnutrition, stunting, India, rural

## Abstract

Stunting is a major unresolved and growing health issue for India. There is a need for a broader interdisciplinary cross-sectoral approach in which disciplines such as the environment and health have to work together to co-develop integrated socio-culturally tailored interventions. However, there remains scant evidence for the development and application of such integrated, multifactorial child health interventions across India’s most rural communities. In this paper we explore and demonstrate the linkages between environmental factors and stunting thereby highlighting the scope for interdisciplinary research. We examine the associations between household environmental characteristics and stunting in children under 5 years of age across rural Rajasthan, India. We used Demographic and Health Survey (DHS)-3 India (2005–2006) data from 1194 children living across 109,041 interviewed households. Multiple logistic regression analyses independently examined the association between (i) the primary source of drinking water, (ii) primary type of sanitation facilities, (iii) primary cooking fuel type, and (iv) agricultural land ownership and stunting adjusting for child age. The results suggest, after adjusting for child age, household access to (i) improved drinking water source was associated with 23% decreased odds [odds ratio (OR) = 0.77, 95% confidence interval (CI) 0.5–1.00], (ii) improved sanitation facility was associated with 41% decreased odds (OR = 0.51, 95% CI 0.3–0.82), and (iii) agricultural land ownership was associated with 30% decreased odds of childhood stunting (OR 0.70, 95% CI 0.51–0.94]. The cooking fuel source was not associated with stunting. Our findings indicate that a shift is needed from nutrition-specific to contextually appropriate interdisciplinary solutions, which incorporate environmental improvements. This will not only improve living conditions in deprived communities but also help to tackle the challenge of childhood malnutrition across India’s most vulnerable communities.

## Introduction

Childhood undernutrition is a global health priority for sustainable development. Despite persistent efforts the global nutritional targets for children under 5 years of age (U5) remains to be met. In 2017, an estimated 150.8 million children U5 were stunted (low height-for-age), while globally another 50.5 million children were wasted (low weight-for-height) [1]. Contributing to almost one-third of the world’s childhood chronic undernutrition burden with 46.6 million U5 stunting [2], India is the priority target to set the pace of progress towards a better-nourished world.

The World Health Organization (WHO) recognises the first 1000 days as a critical window of opportunity during which timely interventions can have a measurable and lasting impact on health and nutrition [3]. The lasting impact of malnutrition during this critical period of development transcends generations. These include long-term effects on cognitive development, school achievement [4], adult economic productivity, maternal reproductive outcomes [5] and risk for obesity and non-communicable diseases [6].

Stunting is the result of chronic malnutrition and reflects the interaction between intergenerational socio-economic, cultural–behavioural and environmental risk and protective factors [7]. The first 1000 days are exceptionally sensitive to environmental conditions because of ‘greater susceptibility to infections’, ‘high sensitivity to programming effects’ and ‘full dependence on others for care, nutrition, and social interaction’ [8]. A child’s household and immediate surroundings represents one of their earliest exposures to the extrauterine environment. There is a complex myriad of broader contextual environmental and social–cultural factors associated with feeding practices for children, which in turn influences the nutritional status of children and hence stunting. Some of the household factors that have been evidenced, so far, to positively impact nutrition in the U5s in India include:

1. Caste and household economy – In rural India, social caste and household economy have been identified as important factors in studies that have focused on stunting. Stunting rates have been found to be significantly higher among children from low-income families and/or from households identified as belonging to Scheduled Castes or Tribes (59%), compared with middle- and high-income families (33%) [9];
2. Education – Education, especially women’s education, has been found to influence nutrition in low- and middle-income countries [10]. Illiteracy influences an individual’s informed decision-making, personal empowerment and community participation in health initiatives and the influence has been found to be higher in rural areas [11];
3. Improved water, sanitation and hygiene practices – Access to safe water, adequate sanitation and hygiene may reduce the risk of diarrhoeal morbidity, parasitic infection and environmental enteropathy [12] and help ameliorate risk of stunting [13]. Improved water access [14] and sanitation practices [15,16] have also been found to be independently associated with positive nutritional outcomes in urbanised states;
4. Cooking fuel exposure – The exact relationship between cooking fuels and nutrition is not clear. However, access to clean fuels [17] have been found to be associated with positive nutrition outcome in children. A growing body of evidence suggests a link between indoor use of biomass fuels (e.g. wood, agricultural, animal waste) and stunting, compared with energy efficient fuels such as liquefied petroleum gas (LPG)/natural gas or electricity [18]. The smoke from biomass combustion produces air pollutants implicated in recurrent respiratory infections and faltered growth [19] with women and children from rural and peri-urban communities at higher risk due to longer periods of exposure [20];
5. Agriculture – Agricultural land ownership may provide food security and adequate dietary intake to protect against stunting. However, few studies have examined the influence of agricultural land ownership on stunting in rural households. Here, rural tribes may be differentially vulnerable to food insecurity and thus to stunting due to seasonal isolation (i.e. lack of grazing land) and economic deprivation (i.e. high cost of treatment for diseased animals [21]). A recent review of the impact of agricultural interventions on nutritional outcomes between 2000 and 2014 revealed that nutrient intake and ultimately nutritional outcomes can be improved by the production of targeted nutrition-rich crops, homestead gardens and diversification of the production system [22].

India’s first National Nutrition Mission (NNM), launched in 2018, focuses on multisectoral convergence to comprehensively impact the environment to improve nutrition. Convergence to improve childhood nutrition has so far been achieved through close coordination between two programmes – the Integrated Child Development Services (ICDS) and the National Rural Health Mission (NRHM) [23]. However, while stunting rates are well documented across India, the environmental and social determinants affecting feeding practices of households in rural communities are not clearly understood [24], which leaves the NNM uninformed of specific convergence interventions in the context of the NRHM and the ICDS. This is an important omission and may explain why current nutrition-specific initiatives have failed in the past to address the growing global health issue of stunting across rural India.

India’s National Family Health Survey (NFHS) provides data from representative samples at national and state levels on a comprehensive list of domains that include health, nutrition, fertility, mortality and family planning with a focus on women and children. The household interviews also provide critical estimates on different household characteristics. In recent years, the NFHS has been the basis of claims on successes of the NRHM [25]. The value of using NFHS data to improve nutritional outcomes through the ICDS and the NRHM has been recognised [26]. Conducted in 2005–2006, NFHS-3 provides a profile of important baseline statistics on the association of different factors related to larger household environments and nutrition at the commencement of NRHM, launched in April 2005, which is why we are basing our study around this database.

Therefore, in this study, we aimed to identify which household environmental characteristics are associated with childhood stunting as a first step towards better informing current national strategies. Specifically, we examine the associations between: (i) main drinking water source, (ii) main type of sanitation facilities, (iii) main cooking fuel and (iv) ownership of agricultural land and stunting in children U5 from the NFHS-3 data. In order to provide evidence for underserved and typically undernutrition-affected states, we limited the analysis to Rajasthan which is a landlocked state in North-Western India, characterised by large numbers of tribal groups (75% of individuals live in rural areas, compared with 25% in urban areas) and low female (42%) relative to male (76%) literacy rates [27].

## Method

### Data source

We examined the Demographic and Health Survey (DHS)-3 carried out by the International Institute of Population Sciences (IIPS) in 2005–2006 [2]. Briefly, a stratified multistate cluster sampling method identified a nationally representative sample of India’s population living in both urban and rural areas in 29 states. The data collection was carried out by the IIPS between November 2005 and August 2006 and included data on 515,597 individuals from 109,041 interviewed households across India. The three core questionnaires of the DHS-3 are the Household Questionnaire, the Women’s Questionnaire and the Men’s Questionnaire and pertain to indicators in the areas of population, health and nutrition. In the current study, we examine data from the Household Questionnaire for the state of Rajasthan in India, which includes the following information:

- Household schedule: age, sex, relationship to the head of the household, education, parental survivorship and residence, and birth registration.
- Household characteristics: drinking water, toilet facilities, cooking fuel and assets of the household.

### NFHS-3 data collected and study indicators

All interviews and anthropometric measurements were collected as part of the DHS-3 following the guidelines set out by the IIPS. Each household respondent was invited to provide informed consent with parents or guardians providing consent for infants and children prior to the interviews and assessments. The field interviewers and anthropometrists were from local non-government organisation (NGO) partners and were trained before data collection. The performance of field staff during data collection was continuously monitored by supervisors and quality control teams who rechecked some of the data the following day to ensure reliability.

### Household environmental characteristics

DHS-3 interviews were carried out using structured questionnaires. During the Household Questionnaire, respondents could select only *one* of the following sub-categories pertaining to each household category:

- Main drinking water source: piped into dwelling, piped into yard, public tap, borehole, protected well, unprotected well, unprotected spring, groundwater, rainwater and tanker truck or cart.
- Main sanitation facility: flush to piped sewer system, flush to septic tank, flush to pit latrine, flush elsewhere, and ventilated pit latrine, pit latrine with slab, pit latrine without slab, no facility/field/bush, and dry toilet or other.
- Main cooking fuel source: LPG/natural gas, kerosene, charcoal, wood, straw/shrubs/grass, agricultural waste and animal waste.
- Agricultural land ownership: yes or no.

### Anthropometry

In the original study from which the data was generated, the length of each child per household (at 0–23 months) was measured in a recumbent position to the nearest 0.1 cm using a measuring board. The height of each child (>24 months) was measured in a standing, upright position to the nearest 0.1 cm using a vertical board with a detachable sliding headpiece.

### Other (confounding) variables

The confounding variable age was selected on the basis of three conditions [28]:

1. Age was associated with both stunting and different explanatory factors, including feeding practices (e.g. infants and children have predominantly different feeding practices); risk of infection (the risk of ingesting bacteria from human and animal sources via open defecation in children who start to grow, crawl, walk, explore and put objects in their mouths increases);
2. Age was unequally distributed among the different outcome groups;
3. Age cannot be an intermediary step in the relationship of any of the suspected independent variables with stunting, as age cannot have a predictor.

The child’s age was given by the parent, guardian or other household respondent at the time of administering the DHS-3 questionnaire. As it can be difficult for rural households to accurately estimate a child’s age without a birth certificate or vaccination card, DHS-3 field staff used a local events calendar to determine the month and year of birth of the case. The child’s age in months was calculated using the country’s month code for the date of the interview, minus the country’s month code for the date of birth of the child.

As the feeding practices differ between children aged 0–6 months and 6–24 months, this study follows the WHO [29] standard by analysing the following age categories: <6 months, 6–24 months and 24–60 months.

No other non-modifiable characteristic, for example, sex of the child or caste of the household, etc., met the criteria for confounding variables.

### Statistical analyses

We analysed the data in SPSS 25 (IBM SPSS Statistics, Portsmouth, UK) [30]. First, only children under 5 years of age (herein cases U5) with available information on age, sex and height were retained in the dataset. The final number of cases with available data (n = 1194) formed the basis of the analyses. Second, stunting indices were calculated as per the WHO child growth standards using the age and height data collected and defined as height-for-age (HAZ) z-scores less than two from the median HAZ of a reference population [29]. Third, improved drinking water source was dichotomised into *improved* (piped into dwelling, piped into yard, public tap, borehole and protected well) versus *unimproved* as per WHO [31] guidelines. As reported elsewhere [15], sources of sanitation facilities were also dichotomised into *improved* (including flush to piped sewer system, flush to septic tank, flush to pit latrine, flush elsewhere, ventilated pit latrine and pit latrine with slab) versus *unimproved*. Improved cooking fuel was dichotomised as *improved* (LPG/natural gas and kerosene) versus *unimproved* [32]. Fourth, descriptive statistics were used to examine the distribution of the full range of variables, that is, household characteristics and stunting. Lastly, a cross-tabulation with chi-square (χ^2^) analyses were run as the main analyses. Where a significant association was found, a multiple logistic regression model was used to independently examine the association between household characteristics and stunted cases (0 = not stunted; 1 = stunted) adjusting for infant age category as a potential confounder. Household characteristics were included as the independent variables and stunting was included as the dependent variable. We used logistic regression to inform the predictors of stunting because logistic regression is a widely used technique for modelling predictors of binary outcomes and has been applied to the same survey data to predict the effect of biomass fuels on mother-reported child size [33]. The odds ratio (OR) and corresponding 95% confidence intervals (CI) were estimated with statistical significance defined as *p* ≤ 0.05. Use of similar estimates would allow better synthesis of evidence and would provide evidence comparability.

## Results

### Descriptive statistics

#### Demographic characteristics

The mean age (± SE) of cases U5 in the analyses was 29.9 ± 0.51 months, 53% were male and 81% belonged to Scheduled Castes. Approximately 44.5% of the sample were stunted. Stunting cases significantly differed by age category *F*(1) = 51.35, *p* ≤ 0.001, all levels were significant. Hence, only case age was adjusted for in the following regression analyses (Table 1).

**Table 1.** Characteristics of cases under 5 years of age included in the sample.

Demographic characteristics	Total sample (n = 1194)	Stunted (n = 532)	Not stunted (n = 662)
Child age (months), M (±SE)	29.98 ± 0.51	33.32 ± 0.67	27.31 ± 0.73
Child sex, n (%)			
Male	636 (53.3%)	285 (53.6%)	351 (53%)
Female	558 (46.7%)	247 (46.4%)	311 (47%)
Caregiver schedule	–	–	–
Caste	976 (81.7%)	425 (79.9%)	551 (83.2%)
Tribe	218 (18.3%)	107 (20.1%)	111 (16.8%)
Stunted (HAZ <−2), n (%)			
No	662 (55.5%)	–	–
Yes	532 (44.5%)	–	–
Stunting (HAZ <−2), M (±SE)	−1.80 (1.76)	−3.34 (0.04)	0.56 (1.18)
Drinking water source, n (%)			
Piped into dwelling	17 (1.4%)	5 (0.9%)	12 (1.8%)
Piped into yard	107 (9%)	50 (9.4%)	57 (8.6%)
Public tap	195 (16.3%)	91 (17.1%)	104 (15.7%)
Borehole	527 (44.1%)	223 (41.9%)	304 (45.9%)
Protected well	18 (1.5%)	8 (1.5%)	10 (1.5%)
Unprotected well	221 (18%)	110 (20.7%)	111 (16.8%)
Unprotected spring	2 (0.2%)	–	2 (0.3%)
Groundwater	35 (2.9%)	16 (3.0%)	19 (2.9%)
Rainwater	28 (2.3%)	7 (1.3%)	21 (3.2%)
Tanker truck	14 (1.2%)	7 (1.3%)	7 (1.1%)
Cart	30 (2.5%)	15 (2.8%)	15 (2.3%)
Sanitation facility, n (%)			
Flush to pipe sewer system	1 (0.1%)	–	1 (0.2%)
Flush to septic tank	43 (3.6%)	16 (3.0%)	27 (4.1%)
Flush to pit latrine	27 (2.3%)	4 (0.8%)	23 (3.5%)
Flush elsewhere	1 (0.1%)	1 (0.2%)	–
Ventilated pit latrine	1 (1.1%)	1 (0.2%)	–
Pit latrine with slab	17 (0.4%)	6 (1.1%)	11 (1.7%)
Pit latrine without slab	11 (0.9%)	4 (0.8%)	7 (1.1%)
No facility/field/bush	1088 (9.1%)	498 (93.6%)	590 (89.1%)
Dry toilet	2 (0.2%)	1 (0.2%)	1 (0.2%)
Other	3 (0.3%)	1 (0.2%)	2 (0.3%)
Cooking fuel source			
LPG/natural gas	33 (2.8%)	9 (1.7%)	24 (3.6%)
Kerosene	4 (0.3%)	1 (0.2%)	3 (0.5%)
Charcoal	4 (0.3%)	2 (0.4%)	2 (0.3%)
Wood	1021 (85%)	455 (85.5%)	566 (85.5%)
Straw/shrubs/grass	71 (5.9%)	33 (6.2%)	38 (5.7%)
Agricultural waste	32 (2.7%)	13 (2.4%)	19 (2.9%)
Animal waste	29 (2.4%)	19 (3.6%)	10 (1.5%)
Agricultural land ownership			
No	224 (18.8%)	116 (21.8%)	108 (16.3%)
Yes	970 (81.2%)	416 (78.2%)	554 (83.7%)

HAZ, height-for-age z-scores.

Of the 1194 cases, 72.3% of cases belonged to families reported as using an improved main source of drinking water source, with a borehole as the main source of drinking water (44.1%). Only 7.6% belonged to families that used improved sanitation facilities, and 91% used no sanitation facility. Only 3.1% of cases belonged to families reported as using an improved source of cooking fuel, with biomass fuel (wood) as the commonest source (85%). Lastly, 224 cases (18.8%) belonged to families reported as owning agricultural land, whilst 970 cases (81.2%) belonged to families that did not own agricultural land (Table 1). However, the proportion of no land ownership was significantly higher in stunted children (*p* < 0.05) compared to non-stunted.

### Results from main analyses

#### Drinking water source and stunting outcome

Results from the χ^2^ analyses are reported in Table 2. An unadjusted logistic regression model reported drinking water did not predict stunting outcome (unadjusted OR = 0.78, 95% CI 0.60 to 1.02, *p* = 0.72). There was a significant relationship between drinking water source and stunting when controlling for age category (adjusted OR = 0.77, 95% CI 0.58 to 1.00,* p* = 0.05), with 23% decreased odds of stunting for children consuming water from improved sources in comparison to those who use unimproved sources (Table 3).

**Table 2.** OR and χ^2^ for household characteristics on stunting (HAZ <−2) standardised coefficients and CIs.

Household characteristics (n = 1194)	OR	χ^2^
Drinking water source		
Unimproved	1[Reference]	3.24 (1)
Improved	0.78 (0.60–1.02)	
Age category		
0–5 months		
Unimproved	1[Reference]	0.24 (1)
Improved	0.76 (0.26–2.21)	
6–23 months		
Unimproved	1[Reference]	6.27 (1)*
Improved	0.53 (0.32–0.87)	
24–59 months		
Unimproved	1[Reference]	0.27 (1)
Improved	0.91 (0.65–1.27)	
Sanitation facilities		
Unimproved	1[Reference]	7.87 (1)**
Improved	0.51 (0.32–0.82)	
Age category		
0–5 months		
Unimproved	1[Reference]	1.39 (1)
Improved	2.32 (0.55–9.67)	
6–23 months		
Unimproved	1[Reference]	1.54 (1)
Improved	0.58 (0.24–1.37)	
24–59 months		
Unimproved	1[Reference]	8.96 (1)**
Improved	0.40 (0.22–0.74)	
Cooking fuel		
Biomass	1[Reference]	4.01 (1)*
Improved	0.50 (0.25–0.99)	
Age category		
0–5 months		
Biomass	1[Reference]	1.11 (1)
Improved	0.84 (0.77–0.91)	
6–23 months		
Biomass	1[Reference]	0.44 (1)
Improved	1.13 (0.34–3.80)	
24–59 months		
Biomass	1[Reference]	4.63 (1)*
Improved	0.38 (0.15–0.94)	
Agricultural land ownership		
No	1[Reference]	5.83 (1)*
Yes	0.69 (0.52–0.93)	
Age category		
0–5 months		
No	1[Reference]	0.20 (1)
Yes	0.75 (0.22–2.55)	
6–23 months		
No	1[Reference]	0.24 (1)
Yes	0.87 (0.50–1.51)	
24–59 months		
No	1[Reference]	5.93 (1)*
Yes	0.62 (0.43–0.91)	

^a^χ^2^statistic with degrees of freedom and ORs with 95% CI.

^b^*p < 0.05, **p < 0.01. Models include child age in months.

^c^Improved sources of sanitation facilities: to piped sewer system, flush to septic tank, flush to pit latrine, flush elsewhere, ventilated pit latrine and pit latrine with slab.

^d^Improved drinking water source: piped into dwelling, piped into yard, public tap, borehole and protected well [34] vs. unimproved.

^e^Improved cooking fuel: LPG/natural gas and kerosene.

CI, confidence interval; HAZ, height-for-age z-scores; OR, odds ratio; χ^2^, chi-square.

**Table 3.** Logistic regression models for household characteristics on stunting (HAZ <−2) standardised coefficients and CIs.

Household characteristics (n = 1194)	n	Crude OR (95% CI)	Adjusted OR (95% CI)
Drinking water source			
Unimproved	1105	1[Reference]	1[Reference]
Improved	89	0.78 (0.60–1.02)	0.77 (0.58–1.00)
Sanitation facility			
Unimproved	302	1[Reference]	1[Reference]
Improved	892	0.51 (0.32–0.82)*	0.51 (0.32–0.83)*
Cooking fuel source			
Unimproved	1153	1[Reference]	1[Reference]
Improved	41	0.50 (0.25–0.99)*	0.51 (0.25–1.03)
Agricultural land ownership			
No	224	1[Reference]	1[Reference]
Yes	970	0.69 (0.52–0.93)*	0.70 (0.51–0.94)*

^a^*p < 0.05, **p < 0.01. All adjusted models include child age in months.

^b^Improved sources of sanitation facilities: to piped sewer system, flush to septic tank, flush to ^c^pit latrine, flush elsewhere, ventilated pit latrine and pit latrine with slab.

^d^Improved drinking water source: piped into dwelling, piped into yard, public tap, borehole and protected well [34] vs. unimproved.

^e^Improved cooking fuel: LPG/natural gas and kerosene.

CI, confidence interval; HAZ, height-for-age z-scores; OR, odds ratio.

### Sanitation facility and stunting outcome

Unadjusted models reported a significant association between sanitation facility and stunting (unadjusted OR = 0.51, 95% CI 0.32–0.82, *p* = 0.006). This effect remained after adjusting for age (adjusted OR = 0.51, 95% CI 0.32–0.83, *p* = 0.007), with 41% decreased odds of stunting for children with access to improved sanitation facilities in comparison to those without access (Table 3).

### Cooking fuel source and stunting outcome

There was a significant unadjusted association between cooking fuel and stunting outcome (unadjusted OR = 0.50, 95% CI 0.25–0.99, *p* = 0.49). This association was not significant after adjusting for age (adjusted OR = 0.51, 95% CI 0.25–1.03, *p* = 0.061).

### Agricultural land ownership and stunting outcome

There was a significant association between agricultural land ownership and stunting (unadjusted OR = 0.69, 95% CI 0.52–0.93, *p* = 0.016). This association remained significant after adjusting for age (adjusted OR = 0.70, 95% CI 0.51–0.94, *p* = 0.20), with 30% decreased odds of stunting in children whose family owned agricultural land, compared with children without agricultural land ownership (Table 3).

## Discussion

In this study we found that drinking water source, sanitation facility and agricultural land ownership were associated with reduced stunting odds in children across rural Rajasthan, India. Specifically, reported household use of (i) improved drinking water source was associated with 23% decreased odds of stunting, (ii) improved sanitation facility was associated with 41% decreased odds, and (iii) agricultural land ownership was associated with 30% decreased odds of child stunted growth. Indoor cooking fuel source was not associated with risk of stunting although it did approach trend level.

Overall, our results on the association between sanitation facilities and stunting support findings of other cross-sectional studies in rural India. These studies report that improved sanitation, and particularly handwashing with soap [35], is associated with lower risk of stunting [12,36]. Studies have shown that caregiver self-reported handwashing with soap either after open defecation or before infant feeding offers protective effects for child malnutrition [37] and that personal hygiene offers stronger improvements on stunting than improved household access to water and sanitation alone [15].

Open defecation is widely considered a marker of sanitation and increases the risk of spreading bacterial, viral and parasitic infections including diarrhoea, polio, cholera and hookworm. Diarrhoea causes undernutrition and frequent diarrhoeal episodes reduce resistance to infections [38] and hence has a further impact on stunting [16] and infant mortality [39]. The DHS dataset highlights that 91% of households openly defecate. The 2011 Indian Census found that 70% of rural households do not have access to a toilet or latrine. This differs from figures published by the Government of India’s Swachh Bharat Abhiyan mission where the state of Rajasthan is listed as Open Defecation Free [40].

Nonetheless, India’s widespread open defecation and high population density constitute a double threat. The economic impact of inadequate sanitation is estimated at 6.4% of gross domestic product [38]. Despite rapid economic growth, widespread access to improved water sources and improving literacy rates, affordability coupled with a lack of access to water for the maintenance of toilets is often seen as a barrier for latrine construction. Additionally, there is a need for further work on sanitation service use and personal hygiene practices with local values and beliefs. Open defecation represents a complex interplay between material or educational deprivation and beliefs, values and norms about purity, pollution, caste and untouchability [41]. Parental formal education is reported as being associated with improved health outcomes in U5 children across Indonesia and Bangladesh [4]. These outcomes include protective caregiving behaviours (such as handwashing with soap), complete childhood immunisations, improved sanitation (using lined pit latrines) and decreased odds of stunting. Although mothers are generally the primary caregiver, paternal education is also associated with decreased stunting odds. Education is promoted for both men and women in the Millennium Development Goals (MDGs) which – through improved caregiving practice, job security and income – may shift risk of stunting for India’s most rural and vulnerable communities.

Currently, Indian sanitation policies construct pit latrines by focusing on the ‘demand-side’ approach. In practice, government programmes in rural India have neglected to understand why rural Indian communities openly defecate despite the availability of lined pit latrines. Lined pit latrines require the construction of a concrete lined septic tank for safe storage of faecal matter which then needs to be safely disposed. This has led to construction of more affordable non-lined pit latrines, which potentially contaminate ground water. Hence the costs of construction of safe latrines coupled with the requirements of safe disposal of faecal matter become a barrier for the scale-up of sanitation in rural communities where centralised drainage systems for collection of sewage do not exist. Future rural sanitation programmes must ultimately address affordability and cultural beliefs, values and norms around sanitation and should do so in ways that accelerate progress towards social equality for optimal child growth.

Household access to an improved drinking water source was also associated with stunting, albeit to a lesser extent than improved sanitation access. This corroborates early findings that suggest the potential effects of improved water supply on child growth may be smaller than those of improved sanitation [42]. Overall, there is mixed evidence on the interaction between drinking water source and sanitation on child growth. Longitudinal studies have found positive associations between improved water sources and child linear growth existed only when it was accompanied by improved sanitation and water storage practices [43]. In addition, improved sanitation, rather than improved water source, have been associated with lower risk of stunting in India [12] and the Sudan [44]. More recently, randomised controlled trials in Bangladesh report no long-term benefits of integrated water, sanitation and handwashing, compared with sanitation interventions alone [45]. Further research is required to determine if improved household water supply, its treatment, handling and storage, combined with sanitation practices have synergistic or additive effects on child growth. As noted above, the major pathways of faecal–oral transmission of bacteria may be different for infants compared with adults. Infants who are breastfed receive the majority of their nutrients from breast milk and consume little amounts of drinking water. As children start to grow, crawl, walk, explore and put objects in their mouths, the risk of ingesting bacteria from human and animal sources via open defecation increases. Thus, the number of bacteria they ingest from contaminated water may be small compared with other faecal bacteria ingested in conditions of poor sanitation during developmental exploration.

Our finding that agricultural land ownership is associated with stunted child growth is supported by previous studies assessing land holding and nutritional status in children and adolescents across rural India [46,47]. Our results are unsurprising, in part, given the evidence of associations between household food insecurity and malnutrition. Agricultural land ownership is central to income generation and the provision of affordable, diverse, nutrient-rich foods for rural communities. Children of rural communities often live in close proximity to livestock. These livestock may be an important determinant of child nutritional status by directly influencing nutrient intake. Hence promoting livestock production is a common development strategy. However, the overall influence of livestock ownership is not well understood as very few studies have examined the direct effect on child nutrition. Cross-sectional studies have shown positive effects of livestock ownership on child anthropometry [48]. These studies suggest livestock may serve as a direct source of protein through meat, milk and eggs or indirectly by increasing household income for food expenditure. However, agricultural and livestock ownership may also increase exposure to environmental contamination by virtue of faecal material, which may also lead to stunted growth in children. Further research is necessary to understand the effect of agricultural land ownership on stunting to further inform the development of interventions.

We found no association between cooking fuel source and stunting. Previous research has shown associations between biomass fuels and indoor coal use [17,19] with growth deficiencies. However, it is possible households in our DHS-3 sample may have used a mix of both biomass fuels and clean fuels, which may impact on stunting prevalence compared to the use of only biomass fuels. So far, the negative impact of indoor air pollution has only been strongly implicated in the respiratory system. Biomass fuels release particulate matter, carbon monoxide and other toxins at a much higher rate than kerosene and LPG. Rural households tend to rely more on traditional biomass fuels for their household cooking and heating; burned in simple, inefficient and mostly unvented cooking stoves that generate large volumes of indoor smoke. Children, whose lungs are still developing, are particularly susceptible to irritation and contamination when exposed to biomass fuels and hence may experience excessive respiratory infections. Yet, possible systemic effects on child growth have yet to be explored [49]. Exposure is also usually much greater among women, who tend to do most of the cooking [50], and among young children who often stay indoors and are carried on their mother’s back or lap while she cooks [51]. The combined use of fuel, particulate matter concentrations, frequency of cooking fuel use as well as children’s exposure to cooking fuel were not measured in the DHS-3. Nonetheless, in May 2016 the Indian government began providing below-poverty-line households with LPG connections through the Government of India Ujjawala Scheme and NGOs are currently working to replace traditional cooking stoves with more efficient ones. A permanent transition to clean fuels is perhaps needed and low-cost ventilation solutions may have the potential to temporarily mitigate the impact of biomass fuel burning on adverse child health outcomes. It is also worth noting that barriers to uptake of clean energy range from affordability to perception of food tasting different if another fuel source is used.

### Limitations of the study

The result of this study should be read considering multiple limitations related to the tools used in the survey from which the data were sourced as well as the limitations in statistical inference. The first limitation is data availability as we could only include variables which were captured in the DHS-3 and therefore, potential mediators, moderators or even predictors could have been missed. For example, although improved water source is used as an indicator of higher probability of safe water the DHS-3 data did not include biological indicators of pathogenic contamination that might influence infection risk.

Second, data sources used in this paper did not include local or national interventions within the last five years which may have impacted the nutritional status of children.

Third, the DHS-3 allows one selected answer in each category. Yet, households often have multiple sources of drinking water, sanitation and cooking fuels and the DHS-3 did not collect information related to consumption frequency and quality of drinking water. Additionally, children who are schooled, work and/or use public toilets may be exposed to other environmental pathogenic risks of stunting outside of the home. If so, there is greater cause for concern as our results may underestimate the true associations of environmental determinants and anthropometry. Now the DHS-4 has included more open-ended questions (e.g. ‘how do you clean water?’), which allows for a comprehensive analysis of household environmental practices on subsequent childhood stunting.

Finally, use of cultural memory to record age often creates the bias of marginal clustering of data points near class boundaries. Data was not available to support or refute the chance of this bias.

#### Limitation in inference

First, our study can only infer the factors that can predict the odds of stunting and not the exact pathway of stunting. Furthermore, the data points being of a single time point (originating from one cross-sectional survey), it is not possible to infer causality. For the same reason, any effect of time-varying confounding due to any factor could not be detected.

Second, the effects of household characteristics are likely to be underestimated such that measurement constraints did not permit acknowledgement of any previous or ongoing interventions designed to improve child malnutrition. Third, due to the unbalanced sample with relatively smaller number of data points in some of the improved categories, we could not make any inference on the intercept of the model due to risk of rare event effects on the intercept. Fourth, our analysis cannot infer risk for specific dimensions of categorical variables, where categories have been dichotomised, for example, type of water supply. Within each of the two dimensions of water supply (improved and unimproved), it is impossible to provide an estimate of risk for each dimension (e.g. piped water supply). Finally, as the odds ratio usually provides a wider range of confidence of the point estimate, our inference was limited to comparing the odds and not providing an estimate of probability.

## Conclusions

Assessing the environmental determinants of stunting is a critical step in strengthening the relevant evidence base and developing multisectoral interventions for optimal child growth. Our results lend support to the MDGs, Sustainable Development Goals (SDGs), and the 2016–30 Global Health Strategy and Nutrition Mission, which all emphasise the provision of multisector enablers for optimal nutrition. The onus now is to optimise nutrition outcomes for young children using a framework that is broader than nutrition-specific interventions alone. India’s most vulnerable children need to benefit from interdisciplinary research and integrated, cross-sector interventions that can support environmental improvements in tandem with nutrition-sensitive programmes and awareness campaigns. The provision of basic services such as water and sanitation has a significant role to play in not only improving the environment but also improving child health through reducing stunting. Stunting and child health is dependent on a multitude of factors at the household and community level, which requires concerted efforts by policy makers, researchers and private sector partners. Traditional randomised controlled trials usually inform efficacy of interventions targeting a limited range of factors predicting stunting. Our study highlights the need for more intensive formative research to broaden the scope of identifying the multitude of contextual factors in the real world and add more nuance on the evidence around the effect of different variables, for example, improved water supply and sanitation, whose broader effect could be delineated from the DHS data, and requires targeted research to assess the specific effect of various dimensions of these variables.

## Data Availability

The datasets generated during and/or analysed during the current study are available from the corresponding author on reasonable request.
